# ^1^H NMR metabolic profiling of gastric cancer patients with lymph node metastasis

**DOI:** 10.1007/s11306-018-1344-x

**Published:** 2018-03-06

**Authors:** Hailong Zhang, Longzhen Cui, Wen Liu, Zhenfeng Wang, Yang Ye, Xue Li, Huijuan Wang

**Affiliations:** 1 Joint National Laboratory for Antibody Drug Engineering, Henan Key Laboratory of Cellular and Molecular Immunology, Henan University, Kaifeng, 475004 Henan China; 2 School of Basic Medicine, Henan University, Kaifeng, 475004 Henan China

**Keywords:** Lymph node metastasis, Gastric cancer, Metabolic profiling, Tissue

## Abstract

**Introduction:**

Gastric cancer (GC) is a malignant tumor worldwide. As primary pathway for metastasis, the lymphatic system is an important prognostic factor for GC patients. Although the metabolic changes of gastric cancer have been investigated in extensive studies, little effort focused on the metabolic profiling of lymph node metastasis (LNM)-positive or negative GC patients.

**Objectives:**

We performed ^1^H NMR spectrum of GC tissue samples with and without LNM to identify novel potential metabolic biomarkers in the process of LNM of GC.

**Methods:**

^1^H NMR-based untargeted metabolomics approach combined with multivariate statistical analyses were used to study the metabolic profiling of tissue samples from LNM-positive GC patients (n = 40), LNM-negative GC patients (n = 40) and normal controls (n = 40).

**Results:**

There was a clear separation between GC patients and normal controls, and 33 differential metabolites were identified in the study. Moreover, GC patients were also well-classified according to LNM-positive or negative. Totally eight distinguishing metabolites were selected in the metabolic profiling of GC patients with LNM-positive or negative, suggesting the metabolic dysfunction in the process of LNM. According to further validation and analysis, especially BCAAs metabolism (leucine, isoleucine, valine), GSH and betaine may be as potential factors of diagnose and prognosis of GC patients with or without LNM.

**Conclusion:**

To our knowledge, this is the first metabolomics study focusing on LNM of GC. The identified distinguishing metabolites showed a promising application on clinical diagnose and therapy prediction, and understanding the mechanism underlying the carcinogenesis, invasion and metastasis of GC.

**Electronic supplementary material:**

The online version of this article (10.1007/s11306-018-1344-x) contains supplementary material, which is available to authorized users.

## Introduction

Gastric cancer (GC) ranks as the fifth most common malignant cancer and is the third mortality of cancer around the world. About 951,600 new GC cases and 723,100 associated deaths were reported worldwide in 2012 (Torre et al. 2015; Song et al. 2017). GC is particularly prevalent in East Asia, especially in China and Japan (Torre et al. 2015; Leung et al. 2008). Hence, the economic burden on GC is greater than other countries (Kanavos 2006). GC is a multistep and multifactorial progression, the 5-year survival rate of early stage of GC (EGC), which just invaded the mucosal or sub-mucosal layer, reaches over 90% after surgery (Jung et al. 2014; Wang et al. 2016). However, EGC is difficult to be found because of its asymptomatic. So many patients are diagnosed at an advanced stage with lymph node metastasis (LNM) or remote metastasis. As primary pathway for metastasis, the lymphatic system is an important prognostic factor for GC patients. LNM is a single indicator in GC patients with Borrmann type I (Chen et al. 2007). The 5-year survival rate of GC patients with LNM drops to less than 30% (Chen et al. 2002). In addition, the prognosis of node-negative GC patients is markedly better than that of LNM-positive ones (Hyung et al. 2002). The recurrence rises to 10.6–14.8% in LNM-positive EGC from 2.7 to 4.4% node-negative EGC, even following curative surgical operation with D1 + b or D2 lymph node resection (Lai et al. 2009; An et al. 2010). Therefore, LNM may be a critical factor for assessment of prognosis and therapy of GC (Deng and Liang 2014). The overall recurrence rate in LNM-positive GC patients is obviously higher than that in LNM-negative patients, and the overall survival of LNM-positive GC patients is significantly shorter than that of LNM-negative patients (Nakamoto et al. 2007; Sarela et al. 2003). Although the incidence and mortality rates of GC have declined in many countries with the economic improvements, the significant challenges of understanding and treating of GC continue to exist, especially in the process of LNM.

Cancer has been shown to be a metabolic disease, metabolism disorder is a significant characteristic (Warburg 1956). Metabolomics, a rapidly expanding field of systems biology, aims to detect, identify and quantify as many low molecular weight metabolites as possible in a cellular or biological system at a given time. Metabolites can reflect the minor alteration at the level of genome, transcriptome and proteome. Therefore, metabolomics is increasingly used for identifying biomarkers for the early diagnosis and understanding the underlying mechanism of various cancers (Wang et al. 2013; Li et al. 2014; Zhang et al. 2016). Nuclear magnetic resonance (NMR) and mass spectrometry (MS) are the most common tools to characterize the metabolites in body fluids, tissues and cells using targeted or untargeted studies (Griffin and Shockcor 2004). Typically, untargeted ^1^H NMR metabolic profiling, with the advantages of the relative ease of sample preparation and non-destructive analysis has been a popular technique for metabolomics study in various diseases, including arthritis (Weljie et al. 2007), lung cancer (Rocha et al. 2011), melanoma (Wang et al. 2014). Today, most studies focus on identifying biomarkers or exploring the underlying mechanism of GC (Kuligowski et al. 2016; Ramachandran et al. 2016; Gu et al. 2016). However, little attention has been paid to metabolic profiling of LNM, which is particularly important predictor of survival and recurrence in GC (Kunisaki et al. 2009; Lee et al. 2012).

In this study, we demonstrated the metabolic profiling between GC tissues and normal controls, especially the metabolic changes between LNM-positive and LNM-negative GC patients, using an untargeted metabolomics approach based on ^1^H NMR, coupled with multivariate statistical analyses. We totally identified 33 distinguishing metabolites between GC tissues and normal controls, especially 8 of which performed noticeably well in discriminating LNM-positive from LNM-negative GC patients, including branched-chain amino acids (BCAAs: leucine, isoleucine, valine), glutathione, glycine, betaine, tyrosine and hypoxanthine. To our knowledge, this is the first metabolomics report to identify altered metabolites in LNM-positive GC patients, with the hope of identifying potential biomarkers for early diagnosis, staging and prognostic prediction. We also intended to explore the underlying mechanism of LNM in GC.

## Materials and methods

### Chemical reagents

Distilled water was prepared by Milli-Q purification system. Deuterium water (D_2_O, 99.8%) was bought from CIL (Cambridge Isotope Laboratories, USA). High-performance liquid chromatography (HPLC) grade methanol and HPLC-grade chloroform were bought from Merck (Germany). Trimethylsilylpropionic acid-d4 sodium salt (TSP) was bought from Sigma-Aldrich (USA). All other reagents were of analytic grade in our study.

### Patients and samples collection

The population recruited in this study totally consisted of 80 patients with primary GC, who underwent surgical resection at the West China Hospital of Sichuan University between 2013 and 2014. The study protocol was approved by the Ethics Committee of the West China Hospital and the written informed consents were obtained before samples collection. Part of the dissected specimens was used for pathological diagnosis, and the other part was immediately frozen in liquid nitrogen and stored at − 80 °C. To ensure the accuracy, the patients, who received any radiation, chemotherapy or had dissected lymph nodes less than 15, were excluded from our study. The pathological diagnosis was confirmed by standard hematoxylin and eosin stain, and each dissected lymph node was stained with H and E. All patients were diagnosed for TNM stages according to the 7th edition of the American Joint Commission on Cancer (AJCC).

### Preparation of tissue extraction and ^1^H NMR spectroscopic analysis

The reagents were ice-cold before use, including distilled water, D_2_O, methanol and chloroform. To extract the interesting metabolites, the 100–400 mg frozen specimen was minced in liquid nitrogen, weighted and transferred into a 15 mL centrifuge tube. Four milliliter methanol per gram of specimen and 0.85 mL distilled water per gram of specimen were added into the same centrifuge tube and the mixture was vortexed for 1 min. Then 2 mL chloroform per gram of specimen was added and the sample was vortexed again, followed keeping on ice for 30 min. After centrifuging at 1000×*g* at 4 °C for 30 min, the mixture was separated into three phases, including the bottom lipid phase, the middle protein phase and the top aqueous phase, where the metabolic extraction was contained. Each upper water phase was gently absorbed and transferred into a fresh 1.5 mL centrifuge tube and evaporated under a stream of nitrogen. The dried residue was dissolved in 580 µL of D_2_O, which contained 30 µM phosphate buffer solution (PBS, pH 7.4), providing the deuterium lock signal for the NMR spectrometer, and 0.5 mM sodium (3-trimethylsilyl)-2,2,3, 3-tetradeuteriopropionate (TSP), providing the chemical shift reference (δ0.0) and internal concentration standard. The suspension was vortexed and centrifuged at 12000×*g* at 4 °C for 5 min, and about 550 µL solution was loaded into a 5 mm NMR tube for NMR spectroscopy (Beckonert et al. 2007).

### ^1^H NMR spectroscopy

The ^1^H NMR spectra of each samples were recorded on a Bruker Avance II 600 spectrometer (Bruker Biospin, Germany) at 600.13 MHz and 20 °C. One-dimensional NMR spectrum from each sample was acquired using a standard (1D) Carr-Purcell-Meiboom-Gill (CPMG) pulse sequence to observe metabolite signals and 5 s relaxation delay at the water peak position to suppress the residual H_2_O signal. Sixty four free induction decays (FIDs) were collected into 64 K data points with a spectral width of 12335.5 Hz spectral and an acquisition time of 2.66 s. Therefore, the total pulse recycles delay of 7.66 s.

### NMR data processing

The raw NMR data (FIDs) were manually phased adjusted and baseline corrected using MestReNova-6.1.1–6384 software after referencing to TSP chemical shift at 0 ppm. To reduce the complexity of spectral data before statistical analysis, spectral binning was used for producing a data set with manageable proportion. In our study, the spectral regions at 0.5– 9.5 ppm were segmented into 1800 bins with equal width of 0.005 ppm. The spectral regions 3.37–3.34 ppm (methanol signal), 4.94–4.66 ppm (residual water signal) and 7.84–7.62 ppm (chloroform signal) were respectively excluded. In order to get the real metabolites changes in samples, especially for the low concentration metabolites, the integral values of all bins were divided by the value of reference TSP and then normalized to the weight of the measured GC tissue used for metabolites extraction. Moreover, the normalized data was given the same total integration value for each spectra before multivariate data analysis.

### Multivariate statistical analysis

The normalized NMR data in the form of excel was imported into statistical software SIMCA-P (Version 11, Umetrics, AB) for multivariate statistical analysis, including principal component analysis (PCA), partial least squares-discriminant analysis (PLS-DA), and orthogonal projection to latent structure (OPLS). PCA was firstly performed to see an overview and possible outliers. Then PLS-DA was cross-validated by a permutation analysis (200 times), and the resulting R^2^ and Q^2^ values were calculated. OPLS was performed to get well separation between GC cases and controls. The parameters R^2^ and Q^2^, indicating the interpretability and predictability of the model respectively, were used to evaluate the quality of the model. The coefficient plot was color-coded with the absolute value of coefficients (r) with Matlab scripts, meaning the red colored metabolites being more significant than the blue colored ones (Feng et al. 2011). The ROC analysis was performed using predicted Y values of PLS-DA or the relative expression of the specific metabolites in SPSS 22.0.

To identify the interesting spectrum peaks, the variable importance in the projection (VIP) > 1 was considered responsible for group discrimination, which was analyzed and taken as a coefficient from OPLS models. Moreover, unpaired Student’s t-test (p < 0.05) to the chemical shifts was also used to evaluate the reliability of each metabolite. The identified metabolites were chosen as distinguishing ones, which both met VIP > 1 and p < 0.05. The corresponding chemical shift and multiplicity of the metabolites were identified according to the previous literatures and the Human Metabolome Database (http://www.hmdb.ca/).

## Results

### Study population

Based on NMR, a total of 120 tissue specimens were detected in our study, among which, 80 specimens were the matched tumor and control collected from the same patient (*n* = 40). The clinical characteristics were shown in Table 1. The GC patients were divided into two subgroups, including 40 LNM-negative cases (24 male, 16 female; age range, 39–78 years, mean age, 59 years) and 40 LNM-positive cases (22 male, 18 female; age range, 28–82 years, mean age, 60 years). The remaining ones were normal controls (28 male, 12 female; age range, 28–78 years, mean age, 60 years). The recruited patients were all adenocarcinoma according to histology examination. The TNM stages were determined according to the 7th edition of the American Joint Commission on Cancer (AJCC). The detailed information was presented in Table 1.

**Table 1:** Clinical characteristics of gastric cancer patients and normal controls analyzed by ^1^H-NMR

	LNM-negative GC patients	LNM-positive GC patients	Normal controls
No. of subjects	40	40	40
Age (mean (range))	59 (39–78)	58 (28–82)	60 (28–78)
Gender (male/female)	24/16	22/18	28/12
Lauren type			
Intestinal	18	15	41
Diffuse	9	10	
NA	13	15	
Histology	Adenocarcinoma (40)	Adenocarcinoma (40)	
Pathologic grade			
PD	21	12	
MD	16	26	
NA	3	2	
TNM stage			
I	IA:3; IB:17	IB:1	
II	IIA:10; IIB:7	IIA:1; IIB:9	
III	IIIB:3	IIIA:15; IIIB:9	
IV		IV:5	

*PD* poorly differentiated, *MD* moderately differentiated, *NA* not applicable

### Metabolic profiling of ^1^H NMR spectrum of GC tissues

**Fig. 1:**
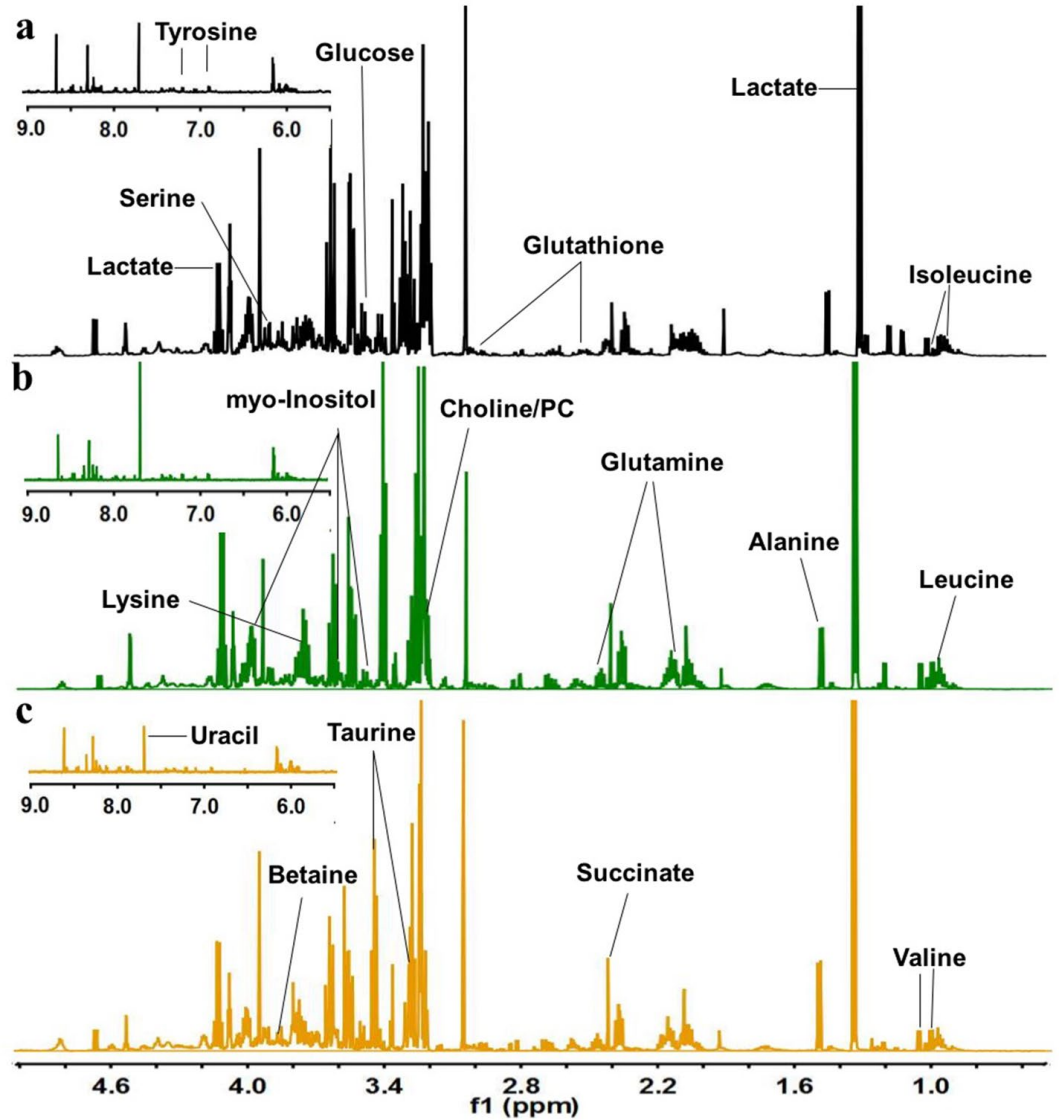
Representative tissue 600 MHz ^1^H NMR spectra. **a** Normal control specimen, **b** LNM-negative GC specimen, **c** LNM-positive GC specimen

Typical NMR spectra of normal control, LNM-negative GC and LNM-positive GC were depicted in Fig. 1a-c, respectively. Resonance assignments were performed by chemical shift and multiplicity of the metabolites according to the previous literatures and the Human Metabolome Database. An overview of visible differences among the three classes was shown in Fig. 1. Most visible spectra were concentrated on the region between 0.5 and 5 ppm, including BCAA, alanine, lactate, glucose, choline/PC, taurine, betaine, and so on. The peaks from 5.5 to 9 ppm were few, including uracil, fumarate, tyrosine and several unknown signals.

### Multivariate statistical analysis of LNM-negative GC specimens, LNM-positive GC specimens and normal controls

**Fig. 2:**
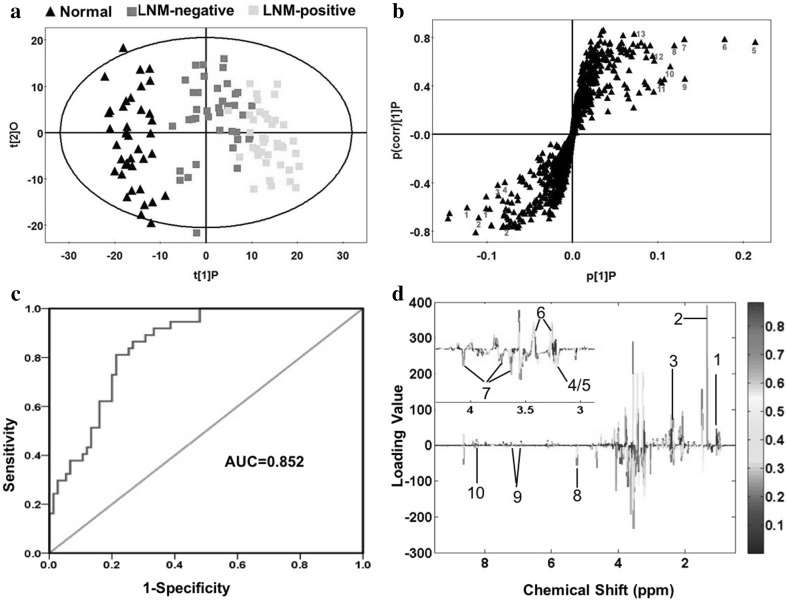
Metabolic profiling between GC tissues and normal controls. **a** OPLS scores plot between the GC tissues and normal controls. Black triangles represent normal controls (n = 40); Green boxes represent LNM-negative GC patients (n = 40); Yellow boxes represent LNM-positive GC patients (n = 40). **b** S-plot of the OPLS model, the variables that lie far away from the center of the plot were assumed to have a greater contribution to the model classification. **c** ROC analysis was performed using the Y-predicted value determined by the PLS-DA model between the GC tissues and normal controls. **d** The color map shows the significance of metabolite variations between the two classes. Peaks in the positive direction indicate the increased metabolites in GC tissues in comparison to normal controls. Decreased metabolites in GC tissues are presented as peaks in the negative direction. 1 BCAA, 2 Lactate, 3 Methylamine, 4/5 Choline/PC, 6 Taurine, 7 myo-Inositol, 8 Glucose, 9 Tyrosine, 10 Hypoxanthine

Firstly, PCA was performed using the raw NMR data. The PCA score plot (2PCs, R^2^X = 0.355, Q^2^ = 0.146) showed clear classification between GC tissues and normal controls (Fig S1a) without any outlier (Fig S1b). Therefore, all of specimens were kept in the further analysis to obtain maximum information. Then OPLS (R^2^X = 0.154, R^2^Y = 0.894, Q^2^ = 0.823) was performed to evaluate variable importance responsible for separation among LNM-negative GC tissues, LNM-positive GC tissues and normal controls. A well separation between GC and controls was shown in Fig. 2a. In the S-plot (Fig. 2b), the variables far away from the center of the plot were assumed to have a greater contribution to the model separation. The model parameters (R^2^ = 0.79, Q^2^ = 0.77) showed good quality of the obtained OPLS model (Fig S1c). A receiver operating characteristic curve (ROC) was carried out using the predicted Y values from OPLS. Area under the curve (AUC) value was 0.852 (Fig. 2c), indicating the OPLS model had good predictive ability. This diagnostic model was just to identify the tissue metabolic biomarkers rather than to replace the established histopathologic diagnostic standard for GC.

We firstly identified 56 metabolites according corresponding chemical shift and multiplicity. Then the inappropriate ones (VIP < 1, or p > 0.05, or both) were excluded. The remaining 30 and 31 significant class-discriminating metabolites (VIP > 1 and p < 0.05) were respectively chosen for LNM-negative and LNM-positive GC cases, which were summarized in Table 2. Among them, 28 distinguishing metabolites were identified both in the two cases. Color-coded coefficient plots showed the change trend of identified differential metabolites responsible for discriminating GC from controls (Fig. 2d). Higher concentration of BACCs (Isoleucine, Leucine, Valine), Lactate, Methylamine and Taurine in GC specimens compared to the controls were illustrated in the positive quadrant signals. On the other hand, lower concentration of Choline/PC, Taurine, myo-Inositol, Glucose, Tyrosine and Hypoxanthine in GC specimens were represented in the negative quadrant signals.

**Table 2:** Differential tissue metabolites among LNM-negative GC patients, LNM-positive GC patients, and normal controls

Metabolites	Chemical shift (ppm, multiplicity)^a^	LNM-negative versus normal controls			LNM-positive versus normal controls		
		VIP^b^	P^c^	FC^d^	VIP^b^	P^c^	FC^d^
Isoleucine	0.945(t)	2.050	0.002	1.058	1.666	0.418	1.329
	1.015(d)	2.318	2.97E-12	1.710	2.539	1.92E-20	2.144
Leucine	0.965(t)	1.725	0.395	1.127	2.250	0.044	1.290
Valine	0.995(d)	1.814	0.327	1.052	2.238	3.06E-04	1.203
	1.045(d)	2.454	0.635	1.025	2.617	0.002	1.167
Lactate	1.33(d)	2.071	4.80E-04	1.193	2.429	2.52E-05	1.219
	4.11(q)	1.675	0.005	1.140	1.953	1.99E-04	1.165
Threonine	1.33(d)	2.071	4.80E-04	1.193	2.429	2.52E-05	1.219
	4.24(m)	2.221	3.52E-04	1.120	1.803	9.71E-05	1.117
Alanine	1.48(d)	2.563	6.27E-06	1.297	2.521	1.23E-07	1.423
Citrulline	1.57(m)	1.134	0.033	0.626	1.565	0.002	0.479
VLDL: –CH_2_–CH_2_–CH_2_O	1.58(br)	1.681	0.006	0.596	1.786	0.005	0.617
N-acetyl glycoprotein	2.05(s)	1.581	0.011	1.245	1.841	0.063	1.110
O-acetyl glycoprotein	2.065(s)	2.520	2.41E-05	1.211	2.340	0.002	1.136
Acetic acid	2.075(s)	2.409	1.23E-23	2.012	2.441	3.21E-26	1.993
Glutamine	2.14(m)	2.204	1.16E-09	1.318	2.479	4.97E-12	1.356
	2.455(m)	2.117	0.057	1.110	2.319	0.031	1.129
	3.77(m)	2.228	3.79E-08	1.216	2.457	1.03E-10	1.260
d-ribose	2.235(s)	1.168	0.004	0.673	1.364	4.33E-04	0.611
Acetone	2.235(s)	1.168	0.004	0.673	1.364	4.33E-04	0.611
Lipid, –CH_2_–C=O	2.26(br)	1.736	0.001	0.814	1.674	2.33E-04	0.809
Pyruvate	2.375(s)	2.537	1.13E-13	1.854	2.399	6.86E-18	2.002
Succinate	2.405(s)	2.334	0.009	1.172	2.305	0.002	1.369
Glutathione	2.555(m)	2.592	1.76E-12	1.537	2.464	1.31E-12	1.618
	2.97(m)	2.429	1.28E-07	1.461	2.320	6.74E-11	1.659
Methylamine	2.595(s)	2.329	1.58E-12	3.554	2.315	2.51E-15	4.335
Choline	3.2(s)	1.097	0.003	0.617	0.870	0.002	0.600
PC (phosphochline)	3.21(s)	1.451	2.06E-05	0.646	1.455	9.32E-06	0.647
Trimethylamine-N-oxide	3.27(s)	1.794	9.61E-12	1.899	1.911	5.37E-20	2.150
Taurine	3.27(t)	1.794	2.87E-11	1.628	1.911	1.02E-16	1.755
	3.425(t)	1.730	2.31E-11	2.562	1.577	4.20E-22	2.801
myo-Inositol	3.535(dd)	2.228	4.75E-11	0.588	2.223	9.90E-28	0.506
	3.63(t)	1.806	7.32E-10	0.716	1.980	7.55E-15	0.667
	4.065(m)	2.035	6.46E-07	0.666	2.144	1.23E-09	0.629
Glucose	3.535(dd)	2.228	1.83E-07	0.588	2.223	3.91E-10	0.506
	5.235(d)	2.624	7.32E-10	0.379	2.505	7.55E-15	0.274
Glycine	3.565(s)	2.596	8.76E-14	2.986	2.619	1.79E-21	3.644
Lysine	3.77(m)	2.228	1.42E-13	1.216	2.457	9.45E-27	1.260
Betaine	3.89(s)	2.451	3.79E-08	0.651	2.504	1.03E-10	0.560
Serine	3.975(m)	2.519	0.001	1.157	2.283	0.003	1.120
Uracil	7.54(d)	2.090	5.03E-06	2.261	2.114	1.14E-06	2.380
	5.8(d)	2.282	2.82E-08	4.657	2.316	3.08E-09	5.179
Fumarate	6.52(s)	1.192	0.007	1.002	1.913	0.004	1.287
Tyrosine	6.9(d)	2.607	0.001	1.324	2.716	1.21E-06	1.492
	7.2(d)	2.231	0.002	1.265	2.384	8.06E-06	1.411
Hypoxanthine	8.18(s)	0.539	0.746	0.964	1.040	0.001	0.632
	8.215(s)	0.939	0.027	0.776	1.682	0.000	0.543

^a^Multiplicity: *s* singlet, *d* doublet, *t* triplet, *q* quartet, *dd* doublet of doublets, *m* multiplet

^b^Variable importance in the projection was obtained from OPLS model with a threshold of 1.0

^c^p-value obtained from Student’s t-test

^d^Fold change (FC) was calculated as a binary logarithm of the average mass response (normalized peak area) ratio between LNM-negative versus normal controls or between LNM-positive versus normal controls

### Multivariate statistical analysis of LNM-negative GC specimens and LNM-positive GC specimens

**Fig. 3:**
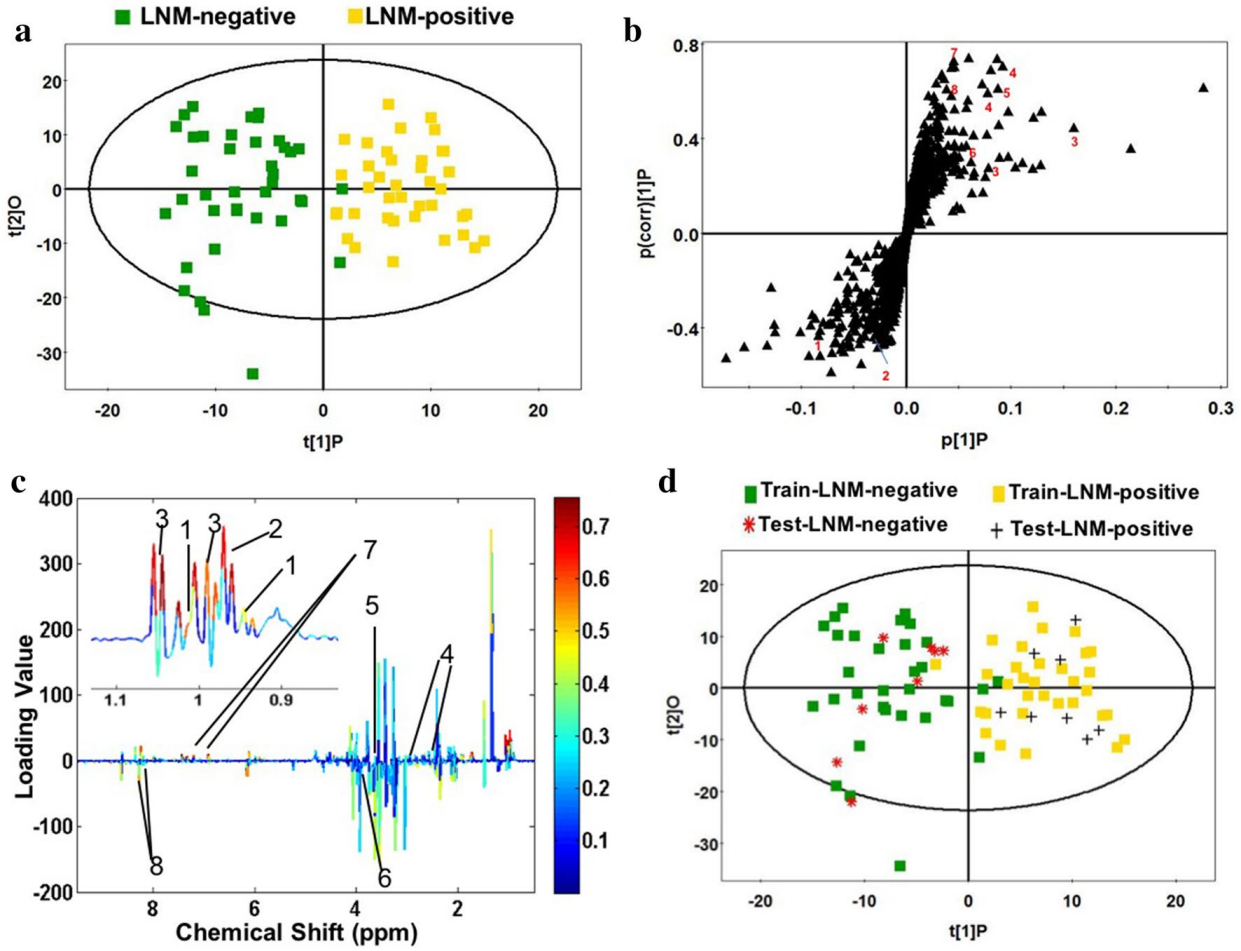
Discriminating plots of LNM-negative and LNM-positive GC patients. **a** Scores plot of OPLS model. Green boxes represent LNM-negative GC patients (n = 40); Yellow boxes represent LNM-positive GC patients (n = 40). **b** S-plot of the OPLS model, the variables that lie far away from the center of the plot were assumed to have a greater contribution to the model classification. **c** The color map shows the significance of metabolite variations between the two classes. Peaks in the positive direction indicate the increased metabolites in LNM-positive GC patients in comparison to LNM-negative GC patients. Decreased metabolites in LNM-positive GC patients are presented as peaks in the negative direction. 1 Isoleucine, 2 Leucine, 3 Valine, 4 Glutathione, 5 Glycine, 6 Betaine, 7 Tyrosine, 8 Hypoxanthine. **d** Scores plot of OPLS prediction model. Eighty percentage of samples were applied to construct the model, and then used it to predict the remaining 20% of samples

To identify the significantly distinguishing metabolites in discriminating between LNM-negative and LNM-positive GC patients, further multivariate statistical analysis was performed. To our knowledge, this is the first study to characterize the metabolic profile of GC with or without LNM, which was useful in identifying potential biomarkers and understanding the underlying molecular mechanism. A good separation between LNM-negative and LNM-positive GC patients was shown in OPLS score plot (R^2^X = 0.121, R^2^Y = 0.75, Q^2^ = 0.485) in Fig. 3a, in which the two cases were mainly distributed at two sides, with only two specimens misclassified. The corresponding permutation analysis (200 times) was displayed in Fig S1d. Model parameters (R^2^ = 0.74, Q^2^ = 0.47) demonstrated the OPLS model was a reliable model with good quality. In the S-plot of the OPLS model (Fig. 3b), the variables that lied far away from the center of the plot were assumed to have a greater contribution to the model classification. To test the predictive ability of established OPLS model in staging GC patients with or without LNM, 80% samples were randomly selected as training set, and the remaining 20% samples were testing set. As a result, the most testing samples were predicted correctly (Fig. 3d), implying LNM was a good indicator for staging GC patients into two subgroups.

**Fig. 4:**
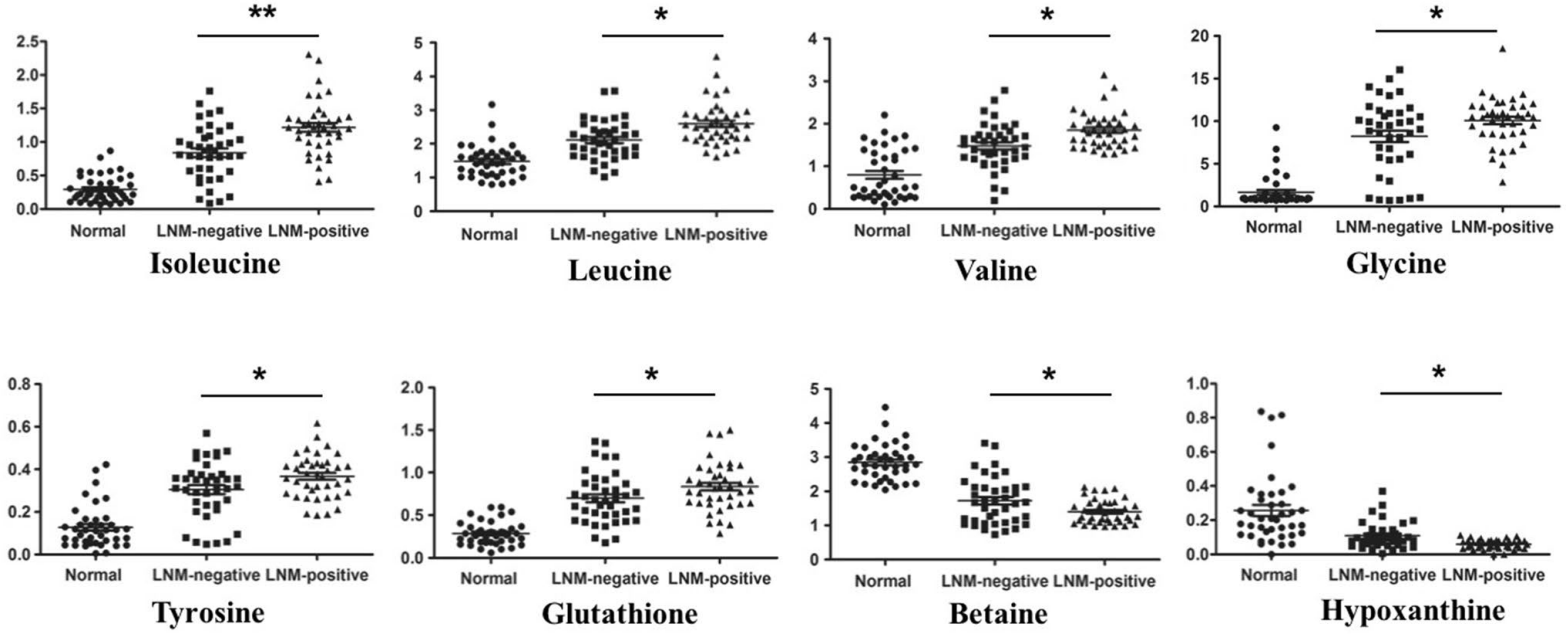
Scatter plots illustrating discrimination among normal controls, LNM-negative and LNM-positive GC patients. The Y axis represents relative abundance of NMR signals (normalized to the total peaks). *p < 0.05; **p  < 0.01 from LNM-positive GC versus LNM-negative GC

To further identified the potential biomarkers in the process of LNM in GC, 8 tissue metabolites were selected with VIP > 1 and p < 0.05, which were listed in Table 3. Compared with that of LNM-negative GC patients, BCAAs, Glutathione, Glycine and Tyrosine were increased in LNM-positive GC patients, on the other hand, Betaine and Hypoxanthine were significantly decreased. In order to clearly show these differences, scatter plots were conducted for illustrating the relative concentration (Fig. 4). The identified differential metabolites between LNM-negative and LNM-positive were also displayed in color-coded coefficient plots (Fig. 3c). Higher concentration of differential metabolites in LNM-positive GC patients compared to the LNM-negative ones were located in the positive quadrant, otherwise, lower concentrations of metabolites in LNM-positive GC patients were shown in negative quadrant.

**Table 3:** Differential metabolites between LNM-negative GC patients and LNM-positive GC patients

Metabolites	Chemical shift (ppm, multiplicity)^a^	LNM-positive versus LNM-negative		
		VIP^b^	P^c^	FC^d^
Isoleucine	0.945(t)	1.059	0.023	1.257
	1.015(d)	3.302	1.73E-04	1.254
Leucine	0.965(t)	2.846	0.010	1.145
Valine	0.995(d)	2.544	0.010	1.143
	1.045(d)	3.086	0.016	1.138
Glutathione	2.555(m)	1.727	0.322	1.052
	2.97(m)	1.524	0.046	1.135
Glycine	3.565(s)	1.622	0.004	1.220
Betaine	3.89(s)	2.081	0.025	0.860
Tyrosine	6.9(d)	3.275	0.045	1.126
	7.2(d)	2.023	0.062	1.115
Hypoxanthine	8.18(s)	1.082	0.002	0.656
	8.215(s)	1.895	0.005	0.700

^a^Multiplicity: *s* singlet, *d* doublet, *t* triplet, *m* multiplet

^b^Variable importance in the projection was obtained from OPLS model with a threshold of 1.0

^c^p-value obtained from Student’s t-test

^d^Fold change (FC) was calculated as a binary logarithm of the average mass response (normalized peak area) ratio between LNM-positive versus LNM-negative

**Fig. 5:**
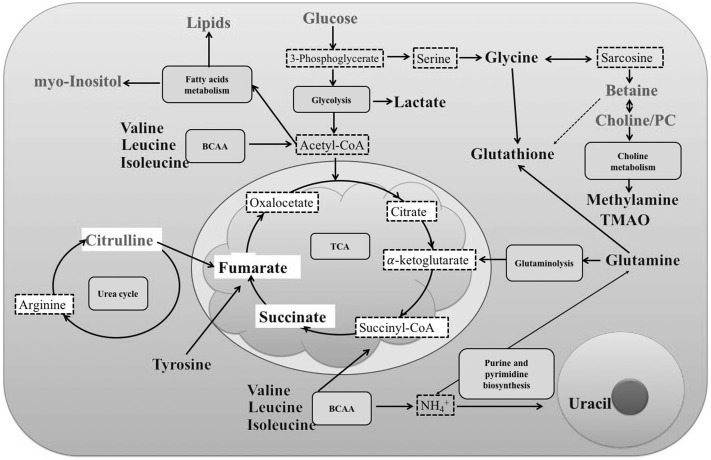
Disturbed metabolic pathways of the relevant metabolites between GC patients and normal controls. Green: lower concentration in GC patients than in normal controls. Red: higher concentration in GC patients than in normal controls

We totally assigned 33 differential metabolites between GC patients and controls in Table 2. After further analyze, eight metabolites were chosen as potential biomarkers for use in discriminating LNM-positive GC patients from LNM-negative ones. To better understand the relationship of the identified metabolites, the related metabolic pathways were conducted in Fig. 5. The altered metabolic pathways included glycolysis, fatty acids metabolism, choline metabolism, glutaminolysis, purine and pyrimidine biosynthesis, urea cycle and TCA. Furthermore, the metabolic pathways including the selected 8 metabolites should be played more attention, such as BCAAs metabolism, Choline metabolism, which may play more important role in the process of LNM in GC.

## Discussion

Lymph node metastasis was an important prognostic factor for GC patients, especially ones with EGC. The patients with LNM-positive had a poorer overall survival compared with those with LNM-negative (Cao et al. 2011; Cheong et al. 2006). In the present study, we analyzed the metabolic profiling of human GC tissue specimens, and identified 33 distinguishing metabolites between GC patients and controls, 28 of which were reported in our previous work (Wang et al. 2016), the other five differently metabolites (Threonine, Alanine, Pyruvate, Taurine and Betaine) were new identified in our study. This might be duo to the difference in the source and quantity of the used specimens. Furthermore, to confirm the potential biomarkers in the process of LNM, further metabolic profiling of LNM-positive or negative patients was performed, 8 differential metabolites were selected with VIP > 1 and p < 0.05. To our knowledge, the study is the first to display the differences of LNM-positive or negative GC patients based on untargeted metabolomics. The identified metabolites may be as the potential factors of diagnose and prognosis of LNM-positive or negative GC patients. Moreover, they also were valuable in understanding the molecular mechanism in the process of LNM.

There were a growing evidences implicating the dysregulation of BCAA metabolism in many disorders like metabolic syndrome, hepatic disease and cancer (Newgard et al. 2009; Kawaguchi et al. 2011; Tonjes et al. 2013). As essential amino acids, BCAAs accounted for over 20–40% of total dietary protein (Brosnan and Brosnan 2006; Harper et al. 1984), which were used to make protein and as nitrogen donors for nonessential amino acid and nucleotide synthesis in proliferating cells, especially in cancer cells. BCAAs were largely transferred into cancer tissues from the blood to supply amino groups (Baracos and Mackenzie 2006), resulting in higher levels of BCAAs in cancer contrast to normal controls. In our previous work (Wang et al. 2016), the levels of BCAAs were also higher in GC tissue specimens. In the present study, BCAAs were not only increased in GC tissues compared with normal controls (Table 2), but also up-regulated in LNM-positive GC patients compared with LNM-negative ones (Table 3, VIP > 1 and p < 0.05). To identify the diagnostic potential of BCAAs between LNM-positive and negative GC patients, ROC curve was performed (Fig S2). AUC was used to assess the potential diagnostic value: AUC (Isoleucine) = 0.745 (95% CI 0.636–0.854), AUC (Leucine) = 0.725 (95% CI 0.614–0.835), AUC (Valine) = 0.741 (95% CI 0.632–0.850), AUC (Combination) = 0.769 (95% CI 0.666–0.873). These results indicated BCAAs may be as predictor in the process of LNM in GC.

BCAA metabolism played a central role in diverse physiological and pathological process, especially in cancer development (Tonjes et al. 2013; Mayers et al. 2014, 2016). The initial step of BCAA catabolism was transferring amino groups to α-ketoglutarate by branched-chain aminotransferase (cytosolic BCAT1 or mitochondrial BCAT2), generating glutamate and the respective α-ketoacids (α-ketoisocaproic acid (KIC), α-keto-*β*-methylvaleric acid (KMV) and α-ketoisovaleric acid (KIV)) (Fig S3a). Through further enzymatic reactions, α-ketoacids were converted to acetyl-CoA and succinyl-CoA, which entered into TCA cycle and were oxidized to supply macromolecule precursors and energy. As an important enzyme, BCAT1 was up-regulated in gliomas, breast cancer and myeloid leukaemia (Tonjes et al. 2013; Zhang and Han 2017; Hattori et al. 2017). Knockdown of BCAT1 repressed the cell proliferation and invasiveness in vitro and inhibited the tumor growth in the xenograft model. In our results, BCAAs were increased in LNM-positive GC patients. Moreover, BCAT1 was also up-regulated in LNM-positive GC tissue specimens compared with controls (Fig S3b). LNM-positive GC patients may need more BCAAs from blood to tissue. The elevated expression of BCAT1 played an important role in initiating the catabolism of BCAAs to serve as macromolecule precursors and energy source. Based on previous literatures and our results, BCAAs may be as a useful diagnostic biomarker of GC patients and distinguishing LNM-positive GC patients from LNM-negative ones. Furthermore, BCAT1 may be as attractive targets for treating LNM-positive GC patients. Certainly, further studies must be performed to investigate the function and mechanism of BCAAs and BCAT1 underlying in GC occurrence and progression.

Cancer cells reprogram metabolism to fulfill their rapid proliferation (Moncada et al. 2012), especially glucose and glutamine supply energy, carbon and nitrogen source for macromolecule synthesis to support cell growth through glycolysis and glutaminolysis (Warburg 1956). In this study, the glucose levels significantly decreased and lactate, as the end product of glycolysis, apparently increased in the LNM-negative and LNM-positive GC specimens compared with controls, which was not surprised because of the well-known Warburg effect (Vander Heiden et al. 2009). While, the Warburg effect was not significantly disturbed between LNM-positive GC patients and LNM-negative ones. Therefore, glycolysis may play a central role in GC tumorigenesis, not in the process of LNM. The similar increased glycolysis was also found in our previous work. Beyond the elevated glycolysis, cancer cells depended more on glutamine (Pan et al. 2015), which can be converted into TCA cycle to generate ATP and intermediates for macromolecule synthesis such as proteins, lipids and nucleotides (DeBerardinis and Cheng 2010). Glutamine was up-regulated in GC tissue specimens compared with controls in our study, which showed GC cells needed more glutamine to support their rapid growth as alternative energy, carbon and nitrogen sources. Therefore, targeting glutamine metabolism for cancer treatment had received increasing attention (Cervantes-Madrid et al. 2017; Dequanter et al. 2017). Although the levels of glutamine didn’t change significantly between LNM-positive GC patients and LNM-negative ones, glutathione (GSH), as a product of glutamine and glutamate metabolism, was increased in LNM-positives ones, which indicated antioxidant capacity was obviously enhanced in LNM-positives GC cells compared with LNM-negative ones. Recently, oxidative stress abnormality has been well acknowledged as a hallmark of cancer. The level of reactive oxygen (ROS) was commonly increased in cancer cells, and some types of cancer cells also demonstrated enhanced sensitivity to ROS. GSH is a key antioxidant, the level of which was elevated in lung, head and neck, ovarian and breast cancer (Gamcsik et al. 2012). The increased level of GSH widely enhanced the antioxidant capacity and resistance to oxidative stress. Therefore, the LNM-positive GC cells with higher level of GSH showed elevated antioxidant capacity and more easily survived from oxidative stress, they were more malignant and metastatic ability. A number of studies have reported GSH would be a reliable marker in solid tumors and hematological cancers (Gamcsik et al. 2012; Kearns et al. 2001). Base on our results and these previous reports, we thought GSH might play an important role in the process of lymph node metastasis of GC.

In our study, choline and betaine were both reduced in GC tissues compared with controls. Especially the level of betaine, as an oxidative metabolite of choline (Ji and Kaplowitz 2003), was lower in LNM-positive GC patients in contrast with LNM-negative ones and controls. Choline and betaine, as major methyl donors in one-carbon metabolism (Zeisel and Blusztajn 1994), may be involved in carcinogenesis, such as colorectal, breast, lung and liver cancer (Lu et al. 2015; Zhang et al. 2013; Ying et al. 2013; Zhou et al. 2017). A recent meta-analysis also demonstrated that the food intake with choline plus betaine (100 mg/day) could lower the cancer incidence by 11% (Sun et al. 2016). Some studies showed one-carbon metabolism was involved in the epigenetic modulation of gene expression and biosynthesis of nucleotides, which may be the cause of being implicated in carcinogenesis (Kim 1999; Pandey et al. 2014). In addition, betaine was involved in the methionine cycle, supplying homocysteine through the transmethylation pathway, which was further used for synthesis of glutathione via transsulfuration (Craig 2004). As mentioned above, GSH could enhance the antioxidant capacity and protect cells from ROS. With the lower level in LNM-positive patients, betaine may be a potential diagnostic biomarker distinguishing LNM-positive GC patients from LNM-negative ones. Our findings together with the previous studies (Sun et al. 2016; Du et al. 2016) proved that betaine might play a critical role in the process of LNM of GC. Therefore, dietary choline and betaine intake with a fixed dose may alleviate GC. While, the actual effect needs to be further investigated.

In addition, we also recognized some limitations in our study. Firstly, our study was performed in a relatively small size in each group. The indication of the robustness of OPLS models was relatively small: R^2^X = 0.121, the maximum fold change is of about 35% (Table 3). Therefore, the interesting potential biomarkers from our study should be verified in the other larger patient cohorts. Secondly, the ^1^H NMR metabolomics platform used in our study was less sensitive than mass spectrometry and only covered a limited number of metabolite targets. which may lost some information about the process of LNM of GC. In addition, two signals of some metabolites were statistically not relevant, such as isoleucine and Hypoxanthine (Table 2), glutathione (Table 3), which should be verified in better metabolomics platform. Lastly, our understanding of these distinguishing metabolites remained at rudimentary levels. More future studies should be focused on elucidating the mechanisms underlying the process of LNM of GC before the clinical application of the candidate metabolites.

## Conclusion

In summary, utilizing untargeted ^1^H NMR spectroscopy combined with multivariate statistical analysis, we globally analyzed the tissue metabolic profiling of GC patients compared with normal controls, especially between LNM-positive and negative GC patients. Totally 33 distinguishing metabolites were identified between GC patients and controls. More importantly, a panel of 8 differential metabolites was selected according to the LNM of GC. According to further validation and analysis, especially BCAAs metabolism, GSH and betaine might be as potential factors of diagnose and prognosis of LNM-positive or negative GC patients. In our knowledge, this is the first metabolomics study focusing on LNM of GC. On the basis of this research, we believed that the identified distinguishing metabolites might show a promising application on monitoring the carcinogenesis, invasion and metastasis of GC. Certainly, future more functional studies and a larger scale of tissue specimen analysis were needed to demonstrate the potential clinical application and the underlying mechanism of GC.

## Electronic supplementary material

Below is the link to the electronic supplementary material.

Supplementary material 1 (DOCX 429 KB)
