# Supplementary material for: 1H NMR metabolic profiling of gastric cancer patients with lymph node metastasis
Source: Metabolomics. 2018 Mar 6;14(4):47. doi: 10.1007/s11306-018-1344-x (PMC5840249; doi:10.1007/s11306-018-1344-x)
Supplement: Supplementary file 1 — Supplementary material 1 (DOCX 429 KB) [file 11306_2018_1344_MOESM1_ESM.docx]

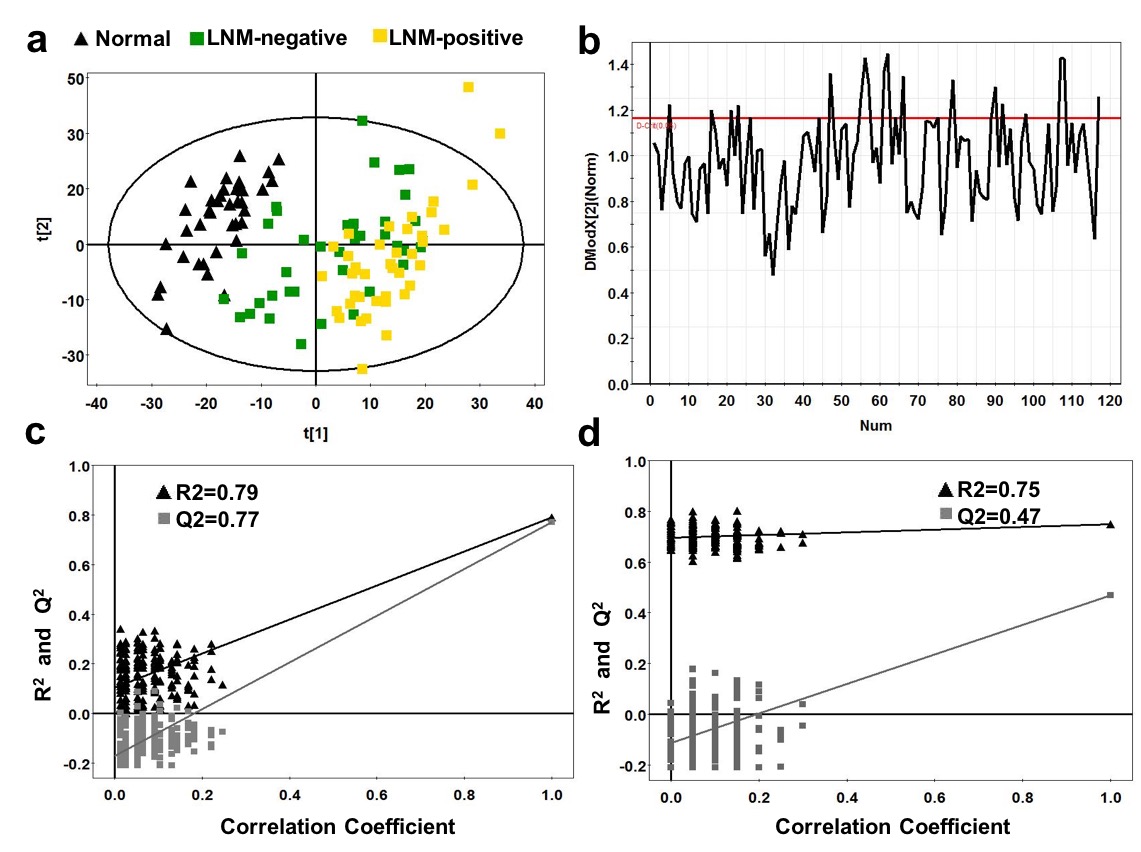


**Fig. S1** Metabolic profiling between GC tissues and normal controls. **a** PCA scores plot between the GC tissues and normal controls. **b** The distance to PCA model showing any outlier. **c** Statistical validation of the corresponding PLS-DA model using permutation analysis (200 times). R^2^ is the explained variance, and Q^2^ is the predictive ability of the model. **d** Statistical validation of the corresponding PLS-DA model using permutation analysis (200 times). R^2^ is the explained variance, and Q^2^ is the predictive ability of the model.


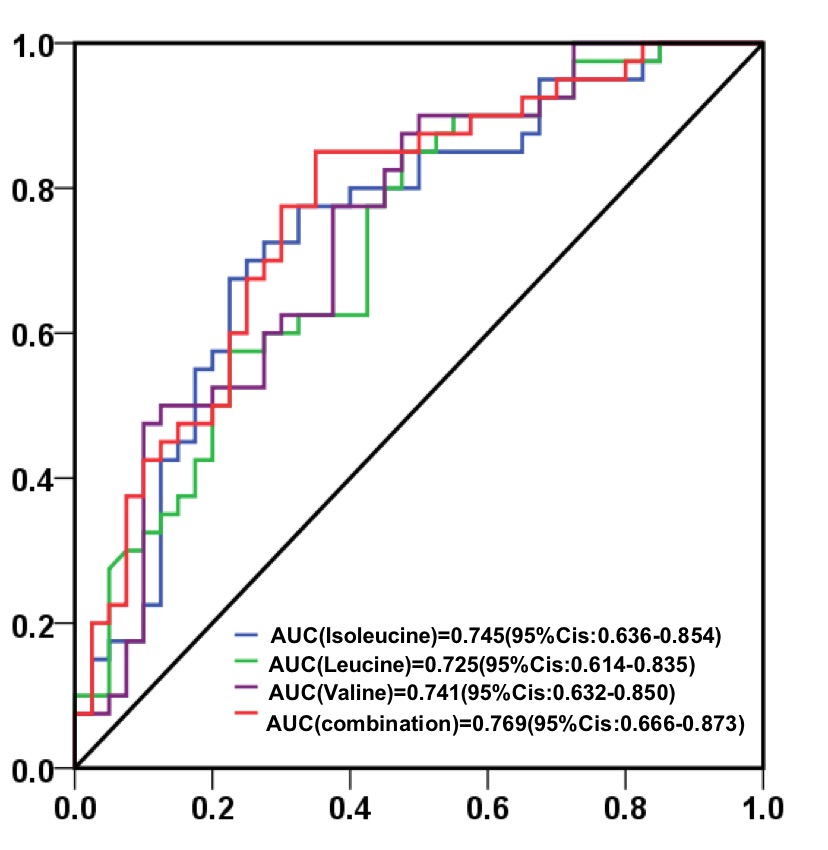


**Fig. S2** Receiver operating characteristic (ROC) curve analysis of BCAAs between GC patients with LNM or not**.** Combination represents isoleucine, leucine and valine.


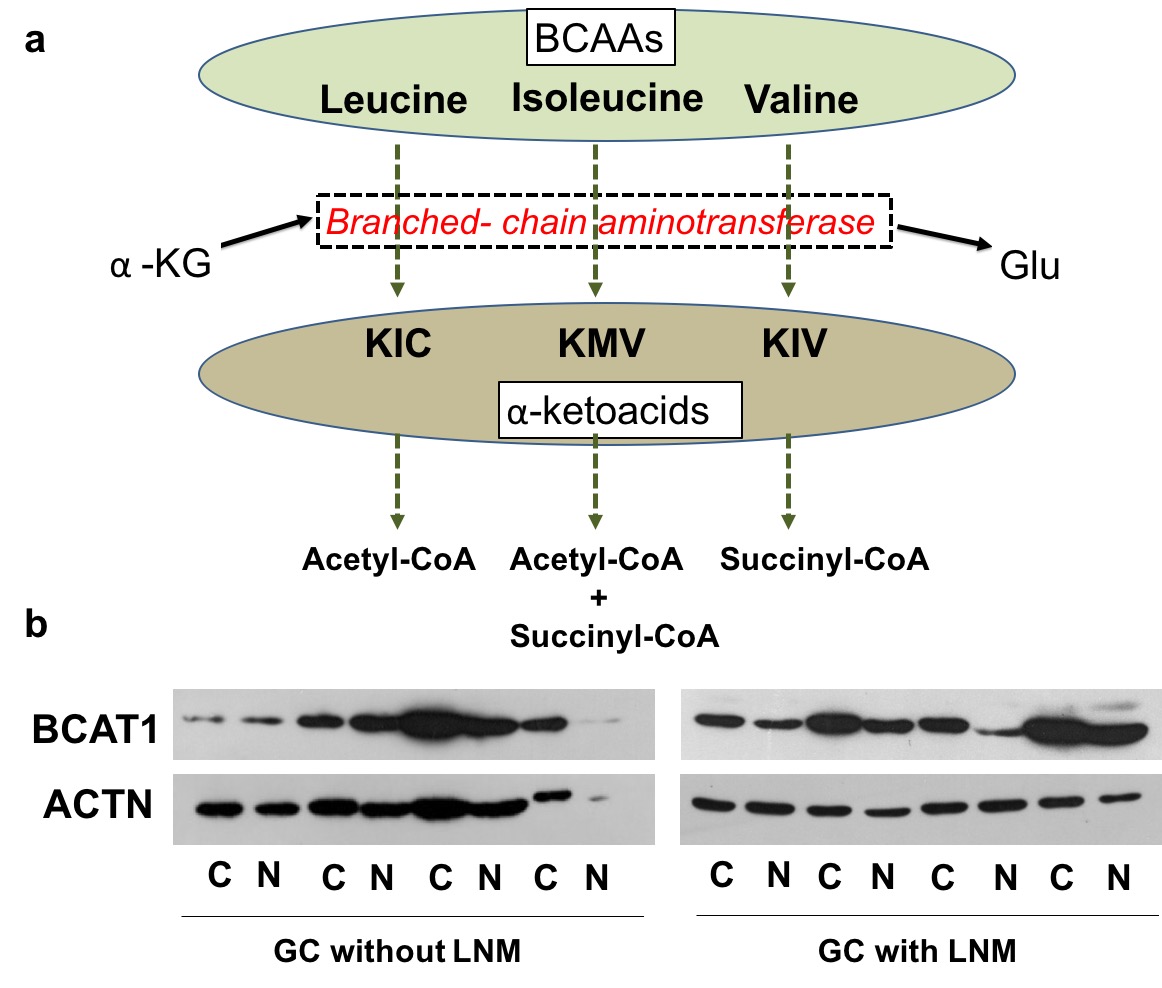


**Fig. S3 a** BCAAs catabolism schematics of the reaction catalysed by BCAT. 𝛼-KG, 𝛼-ketoglutarate; KIC, 𝛼-ketoisocaproic acid; KMV, 𝛼-keto-𝛽-methylvaleric acid; KIV, 𝛼-ketoisovaleric acid. **b** The expression level of BCAT1 in GC tissues by Western blot. BCAT1, branched-chain aminotransferase; C, cancer; N, normal.
